# Relevance of subclinical right ventricular dysfunction measured by feature-tracking cardiac magnetic resonance in non-ischemic dilated cardiomyopathy

**DOI:** 10.1186/s12872-023-03044-x

**Published:** 2023-01-12

**Authors:** J. Urmeneta Ulloa, E. Pozo Osinalde, J. A. Cabrera, M. Recio Rodríguez, I. J. Thuissard-Vasallo, C. Andreu-Vázquez, F. Islas, L. Pérez de Isla, P. Marcos-Alberca, P. Mahía, M. A. Cobos, B. Cabeza, J. L. Rodríguez-Hernández, M. Luaces Méndez, J. J. Gómez de Diego, A. Bustos, J. Pérez-Villacastín, A. de Agustín, V. Martínez de Vega

**Affiliations:** 1 Radiology Department, Hospital Universitario Quirónsalud Madrid, Diego de Velázquez, 1, 28223 Pozuelo de Alarcón, Madrid Spain; 2 Cardiology Department, Hospital Universitario Quirónsalud Madrid, Madrid, Spain; 3 Cardiology Department, Cardiovascular Institute, Hospital Clínico San Carlos of Madrid, Madrid, Spain; 4 Faculty of Biomedical and Health Sciences, European University of Madrid, Madrid, Spain; 5 Cardiology Department, Hospital de San Pedro, Logroño, Spain

**Keywords:** Feature tracking, Right ventricle, Cardiomyopathy, Cardiac magnetic resonance

## Abstract

**Background:**

Right ventricular (RV) dysfunction in patients with non-ischemic dilated cardiomyopathy (NICM) is associated with cardiovascular events. To analyze the feasibility of assessing RV myocardial deformation by feature tracking (FT)-cardiac magnetic resonance (CMR), and its usefulness as a prognostic marker.

**Methods:**

Retrospective study of NICM patients undergoing CMR. Longitudinal FT-RV free wall (LFT-RVFW) and fractional area change (FAC) were obtained. Correlation with standard RV parameters was studied. An association with combined event (heart failure (HF), ICD implantation or cardiovascular death) was assessed using a logistic regression model.

**Results:**

98 patients (64 ± 13 years) were included. Left ventricular (LV) systolic function (LVEF 29.5 ± 9.6%, 47% with LVEF ≥ 30%) and RV (RVEF 52.2 ± 14.6%, 72% with RVEF ≥ 45%). Follow-up of 38 ± 17 months, 26.5% presented at least one admission for HF. An excellent correlation of LFT-RVFW (r = 0.82) and FAC (r = 0.83) with RVEF was evident. No association of RV-FT parameters with prognosis entire study population was found. However, in patients with LVEF ≥ 30%, admissions for HF were associated with lower LFT-RVFW (−21.6 ± 6.6% vs −31.3 ± 10%; p = 0.006) and FAC (36.6 ± 9.6% vs 50.5 ± 13.4%; p < 0.001) values. Similar differences were observed when only patients with RVEF ≥ 45% were considered. An LFT-RVFW cut-off point of -19.5% and FAC of 36.5% showed good prognostic performance. Decreased LFT-RVFW or FAC represented an independent predictor of combined event in patients with LVEF ≥ 30%.

**Conclusions:**

In NICM patients without severe LV dysfunction, decreased values of LFT-RVFW and/or FAC were associated with HF admissions, independently of RVEF.

## Introduction

Non-ischemic dilated cardiomyopathy (NICM) is the cardiac pathology in which left ventricular, or biventricular, dilatation is accompanied by systolic dysfunction, which cannot be explained by abnormal loading conditions secondary to hypertensive heart disease or valvular pathology, as well as the absence of ischemic heart disease [1–3]. The causes include a wide range of genetic and acquired possibilities [1–3], although in many cases it is finally classified as idiopathic.

It is important to diagnose it in early stages of the disease, so that preventive measures and pharmacological treatment can be established to improve the morbidity and mortality of this pathology [4]. Cardiac imaging techniques such as strain speckle tracking in transthoracic echocardiography (TTE) and feature tracking in cardiac magnetic resonance (CMR) have been shown to be able to detect preclinical disease with high reproducibility in trained operators [5–8].

Right ventricle (RV) dysfunction in patients diagnosed with NICM represents a poor prognostic factor in the evolution of this group of patients [9–11]. Analysis of myocardial deformation by echocardiography [12] and CMR [13] has shown added value for risk stratification in patients with NICM. Recently, RV strain by TTE has been used to predict the risk of mortality and hospitalization in patients with dilated cardiomyopathy, defining < − 15.3% as the cut-off point for the greatest development of events in follow-up [14]. However, quantitative assessment of RV function by echocardiography has limitations in relation to its complex geometry and motion, dense trabeculations, and retrosternal position [15, 16], which together with the limited window quality of certain studies makes adequate analysis difficult frequently. An analogous technique for CMR, called feature tracking (FT) [17], of use in recent years, evaluates myocardial deformation from conventional cine sequences, and in the case of the RV overcomes the limitations of echocardiography through a complete visualization of the right ventricular cavity without window limitation.

Our objective is to analyze the feasibility of assessing RV myocardial deformation by FT in NICM. At the same time, to evaluate the added value of longitudinal FT RV free wall (LFT-RVFW) and fractional area change (FAC) calculated by the FT method as a prognostic factor for major cardiovascular events in patients with a diagnosis of NICM.

## Material and methods

### Study population

We retrospectively studied patients with a final diagnosis of NICM, defined according to current recommendations [3], undergoing CMR at our hospital from February 2011 to March 2017. Patients with valvular heart disease or significant coronary artery disease and those with late gadolinium enhancement (LGE) with ischemic pattern were excluded. After ruling out 2 cases due to severe aortic insufficiency as the origin of the ventricular dilatation, 98 individuals were finally included. The left ventricle ejection fraction (LVEF) cut-off point was estimated to be > 55% for patients with preserved LVEF, and < 30% for severely depressed LVEF. Only NICM patients after 3 months optimized medical therapy were taken into consideration.

The patients' personal data and history, as well as medical treatment were obtained from their clinical records. The development of heart failure (HF) events and mortality due to cardiovascular causes, among other variables, were evaluated reviewing medical records. At the same time, the combined event of new admission for heart failure, implantable cardioverter defibrillator (ICD) implantation (secondary prevention, subrogate of malignant ventricular arrhythmia), and mortality was assessed.

This study followed the Declaration of Helsinki for human research and was approved by the Institutional Review Board (code number 19/168-E) on 2019/3/27 at our tertiary level hospital.

### Acquisition and analysis of cardiac magnetic resonance (CMR)

CMR studies were performed on a 1.5 Tesla machine (Signa HDxt® GE) using an 8-channel multi-element surface antenna and ECG synchronization. Cine images were acquired in expiratory apnea and with retrospective ECG protocol using conventional b-SSFP sequences in longitudinal axes with 2-, 3- and 4-chamber views, and in 10–15 contiguous short-axis slices covering both ventricles from base to apex. At around 8–10 min after intravenous administration of 0.2 mmol/kg gadobutrol (Gadovist® 1 mmol/ml, Bayer AG, Germany), LGE images were acquired in the same views as the cine images, using a T1-weighted gradient-echo inversion-recovery (IR) sequence.

Analysis of the studies was performed jointly by a cardiologist and a radiologist with expertise in CMR. The volumes and ejection fraction of both ventricles were obtained from the cine sequences using the disc summation method with specific software (ReportCARD®, GE). Qualitative assessment of the presence and distribution of LGE in inversion-recovery sequences was performed.

**Fig. 1:**
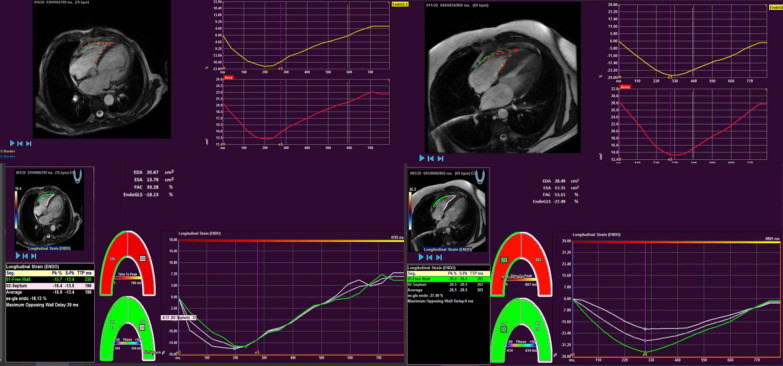
RV free wall tracking feature using CMR. On the left, example of patient with impaired RV dysfunction, decreased LFT-RVFW and FAC. On the right, patient with preserved RV contractile function, normal LFT-RVFW and FAC. *LFT-RVFW* longitudinal feature tracking-right ventricular free wall, *FAC* fractional area change, *RV* right ventricle

FT analysis was performed using QStrain RE® v 2.0 software (Medis) assessing end-diastolic and end-systolic volumes, ejection fraction, FAC and myocardial longitudinal strain parameter from RV long-axis view. The RV endocardial borders were manually traced in one frame, and the contours were automatically propagated by the software to the rest of the cardiac cycle. Finally, the contours were checked and modified manually if necessary. In this way, global RV longitudinal FT values were obtained, subsequently excluding septal values, thus exclusively analyzing those related to the right ventricular free wall, obtaining LFT-RVFW (Fig. 1).

### Statistical analysis

For the descriptive analysis, absolute (n) and relative (%) frequencies were used to express qualitative variables, and mean ± standard deviation (SD) (or median and interquartile range, IQR) to summarize quantitative variables according to their parametric behavior (Kolmogorov–Smirnov normality test).

To compare right ventricular morphofunctional parameters derived from magnetic resonance imaging in relation to admission for heart failure, general and cardiac mortality, and ICD implantation in secondary prevention, the Student t test or Mann–Whitney U test was used, depending on the parametric behavior of the analytical variables. Likewise, the correlation between the FT values was calculated using Pearson's correlation test or Spearman's Rho, depending on the normality or non-normality of the values of these variables.

For parameters of clinical interest, ROC curves were constructed to assess the optimal cut-off point and the sensitivity and specificity for that point, considering that a parameter classified the lesion acceptably when the area under the curve was greater than 0.7.

A logistic regression model was constructed to analyze the effects of right ventricular morphofunctional variables on the combined element (ICU admission, mortality or ICD). All variables with a p-value < 0.1 were included in a multivariate model to identify factors associated with the study variable.

Statistical analyses were performed using SPSS v.25 statistical software tools (SPSS Inc., IL, USA). Statistically significant differences were considered to exist when the p-value was less than 5%.

## Results

### Characteristics of the population

Ninety-eight patients with a diagnosis of NICM were finally included. Invasive coronary angiography had been performed in 87 patients (89%): 66 without coronary lesions, 20 with nonsignificant lesions and 1 with significant lesions that did not justify left ventricular dysfunction. Significant coronary artery disease was ruled out by cardiac computed tomography in 4.1%. None of the CMRs showed LGE with an ischemic pattern.

Regarding baseline characteristics (Table 1A), the mean age was 64 ± 13 years, with 71.4% of the patients being male. In 75.5% of patients, cardiomyopathy was considered idiopathic in nature. Given that many patients were followed up in the HF unit, there was evidence of a high optimization of medical treatment (beta-blockers 92.8%, angiotensin-converting enzyme inhibitors (ACEIs)/angiotensin II receptor blockers (ARBs) 78.5% and antialdosterone drugs 61%, among others).

**Table 1:** General patient baseline characteristics (A) and cardiac magnetic resonance imaging (CMR) findings (B), total sample (n = 98) and according to LVEF (n (%), unless otherwise indicated

	Total	LVEF < 30%	LVEF ≥ 30%	p-value
	(n = 98)	(n = 52)	(n = 46)	
*A. Baseline characteristics*				
Age (mean ± SD), years	64.0 ± 13.0	66.6 ± 12.4	60.7 ± 12.7	**0.023**
Male	70/98 (71.4%)	37/52 (71.2%)	33/46 (71.7%)	0.949
Hypertension	66/98 (67.4%)	35/52 (67.3%)	31/46 (67.4%)	0.993
Diabetes	29/98 (29.6%)	17/52 (32.7%)	12/46 (26.1%)	0.475
Hypercholesterolaemia	36/98 (36.7%)	19/52 (36.5%)	17/46 (37%)	0.966
Smoking	45/98 (45.9%)	23/52 (44.2%)	22/46 (47.8%)	0.722
Etiological diagnosis				N.A
Idiopathic	74/98 (75.6%)	42/52 (80.8%)	32/46 (69.6%)	
Enolic	10/98 (10.2%)	5/52 (9.6%)	5/46 (10.9%)	
Not compacted	5/98 (5.1%)	2/52 (3.8%)	3/46 (6.5%)	
Tachymyocardiopathy	3/98 (3.1%)	1/52 (1.9%)	2/46 (4.3%)	
Peripartum cardiomyopathy	2/98 (2%)	0/52 (0%)	2/46 (4.3%)	
Myocarditis	2/98 (2%)	1/52 (1.9%)	1/46 (2.2%)	
Genetic	1/98 (1%)	0/52 (0%)	1/46 (2.2%)	
Cardiotoxicity	1/98 (1%)	1/52 (1.9%)	0/46 (0%)	
Treatment				
Beta blockers	91/98 (92.9%)	49/52 (94.2%)	42/46 (91.3%)	0.703
ACEIs/ARBs	77/98 (78.6%)	38/52 (73.1%)	39/46 (84.1%)	0.501
Antialdosteronics	60/98 (61.2%)	37/52 (71.2%)	23/46 (50%)	**0.032**
Sacubitril-valsartan	9/98 (9.2%)	5/52 (9.6%)	4/46 (8.7%)	1
Ivabradine	12/98 (12.2%)	5/52 (9.6%)	7/46 (15.2%)	0.399
Furosemide	53/98 (54.2%)	33/52 (63.5%)	20/46 (43.5%)	**0.048**
*B. CMR findings*				
EDV/BSA LV (mean ± SD), ml/m2	133.6 ± 33.4	149.1 ± 32.4	116.1 ± 24.9	**< 0.001**
LVEF (%)	29.5 ± 9.6%	22.0 ± 4.3%	38.0 ± 6.2%	**< 0.001**
EDV/BSA RV (mean ± SD), ml/m2	71.6 ± 20.5	72.0 ± 19.6	71.0 ± 21.7	0.82
RVEF (%)	52.2 ± 14.6	44.9 ± 14.7	60.4 ± 9.2	**< 0.001**
Non-ischemic LGE	38/98 (38.8%)	21/52 (40.4%)	17/46 (37.0%)	0.728
RVEF < 45%	26/98 (26.5%)	24/52 (46.2%)	2/46 (4.3%)	**< 0.001**
Feature tracking-RV				
LFT-RVFW (mean ± SD)	− 23.8 ± 9.9%	− 19.0 ± 6.8%	29.2 ± 10.1%	**0.006**
FAC (mean ± SD)	39.7 ± 16.6%	32.8 ± 15.8%	47.5 ± 13.9%	**< 0.001**
LFT-RVFW < − 18.5%	36/98 (36.7%)	30/52 (57.7%)	6/46 (13.0%)	**< 0.001**
FAC < 32%	33/98 (33.7%)	28/52 (53.8%)	5/46 (10.9%)	**< 0.001**
*C. Mortality*				
Cardiovascular deaths	3/98 (3.1%)	1/52 (2.0%)	2/46 (4.3%)	0.602
Other causes	5/98 (5.1%)	5/52 (9.8%)	0/46 (0.0%)	0.058

*N.A.* not applicable, *EDV* end-diastolic volume, *LVEF* Left ventricle ejection fraction, *RVEF* right ventricle ejection fraction, *LV* left ventricle, *RV* right ventricle, *FW-LFT* free wall-longitudinal feature tracking, *FAC* fractional area change

Patient follow-up was 38 ± 17 months, during which 26.5% required at least one admission for decompensated HF. Mortality due to cardiovascular causes was 3% and due to other causes 5%. An ICD was implanted in 24.5% of patients, 13.3% with resynchronization therapy. For those with TTE control during follow-up (88%), the mean TAPSE was 18.7 ± 5.7% with 11.6% and 5.8% of patients with RV dysfunction and dilatation, respectively.

### Cardiac magnetic resonance imaging

The morphofunctional data of CMRs are compiled in Table 1B. Our population sample had severe left ventricular dilatation (EDV/BSA LV = 133.6 ± 33.4 cc/m^2^) with severe systolic dysfunction (LVEF = 29.5 ± 9.6%). By contrast, the majority had a normal right ventricle in size (EDV/BSA RV = 71.6 ± 20.5 cc/m^2^) and function (RVEF = 52.2 ± 14.6%). Left ventricle (LV) LGE was present in 38.8% of patients, the most frequent pattern being intramyocardial septal enhancement (16.3%). A total of 53% of patients had LVEF < 30%, while 26.5% had RVEF < 45%. Regarding the parameters derived from RV FT, the mean LFT-RVFW of our patients was − 23.8 ± 9.9% with a derived FAC of 39.7 ± 16.6%. A cut-off point of LFT-RVFW of − 18.5% (S = 0.86; E = 0.96), and a FAC of 32% (S = 0.9; E = 0.96) was established to predict RVEF ≥ 45%. A total of 36.7% of patients had LFT-RVFW < − 18.5% and 33.7% with FAC < 32%.

**Fig. 2:**
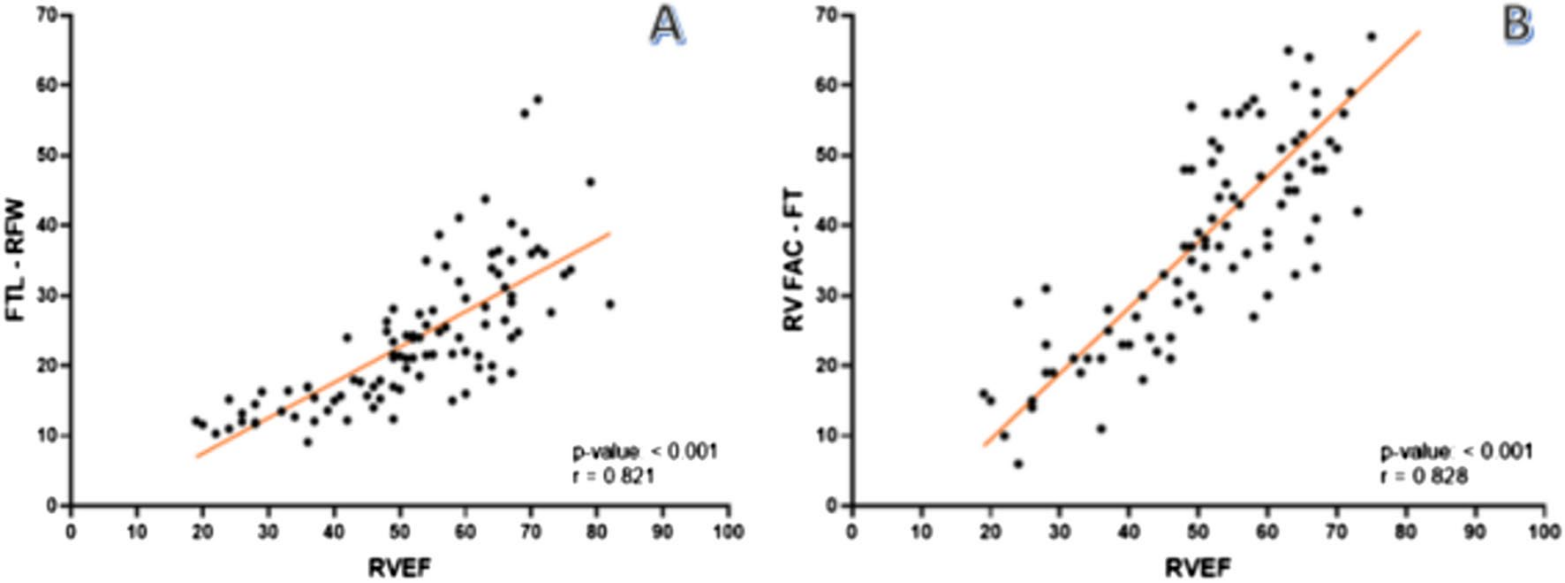
Correlation between LFT-RVFW calculation (**A**) and FAC-FT (**B**) with RVEF calculation by classical disc summation method. *LFT-RVFW* longitudinal feature tracking-right ventricular free wall, *FAC* fractional area change, *RVEF* right ventricle ejection fraction

We found a strong correlation between the calculation of right ventricular systolic function by RVEF using the classic disc summation method and the determination of LFT-RVFW (Spearman's Rho = 0.821, p < 0.001) and FAC (Spearman's Rho = 0.828, p < 0.001) derived from the FT technique (Fig. 2).

When the general population was analyzed, no association of RV myocardial deformation parameters with prognosis was found. However, the subgroup without severe LV systolic dysfunction (LVEF ≥ 30%) had a slightly lower age (Table 1A). In this subgroup, we observed that those admitted for HF had decreased LFT-RVFW values (−21.6 ± 6.6% vs −31.3 ± 10%; p = 0.006). The same association was detected in these patients in relation to lower FAC (36.6 ± 9.6% vs 50.5 ± 13.4%; p < 0.001 ). These findings were consistent in this subgroup of patients for both LFT-RVFW (− 24% ± 10.2% vs -31.2 ± 12.1%; p = 0.007) and FAC (38.3 ± 8.3% vs 51.2 ± 13%; p < 0.001) when selecting only those with preserved RVEF (≥ 45%). Cut-off points of LFT-RVFW of -19.5% and FAC of 36.5% in our population seem to be adequate to detect patients with LVEF ≥ 30%, who are more likely to be admitted for HF (LFT-RVFW: S = 0.94; E = 0.50; FAC: S = 0.92; E = 0.60), even in the presence of RVEF ≥ 45% (LFT-RVFW: S = 0.97; E = 0.44; FAC: S = 0.94; E = 0.56).

On the other hand, although cardiovascular mortality in our sample was very low during follow-up (3.1%) (LVEF < 30%: 1/52; LVEF ≥ 30%: 2/46, Table 1C), probably related to a strict control by the heart failure unit, we observed very low values of LFT-RVFW in patients who died of cardiac causes both in the subgroup of patients with LVEF ≥ 30% (LFT-RVFW, −15.7 ± 4.7% vs −27.5 ± 14.2%; p = 0.036) and in those with LVEF ≥ 30% and LVEF ≥ 45% (LFT-RVFW, −15.7 ± 4.7% vs −27.8 ± 12.5%, p = 0.028).

Univariate analysis was performed in the group of patients with LVEF ≥ 30%, including RVEF, the presence of late enhancement, and the values obtained by FT. Only FAC (OR = 0.890; CI 95% 0.817–0.971; p = 0.008) and LFT-RVFW by this technique (OR = 0.885; CI 95% 0.797–0.984; p = 0.023) were associated with the combined endpoint in this subgroup (Table 2), remaining the FAC association in the multivariate analysis. A decreased LFT-RVFW (− 22.8 ± 7.5% vs − 31.2 ± 10.1%, p = 0.015) and FAC (37 ± 9.2% vs 50.8 ± 13.5%; p = 0.003) represented predictors of combined event, HF admission and mortality in patients with LVEF ≥ 30% in our sample. In those with severely reduced LVEF < 30%, LFT-RVFW was not able to detect differences in the probability of admission for HF, nor were there differences in the combined event, admission for HF or mortality in this subgroup of patients.

**Table 2:** Univariate and multivariate logistic regression models for factors associated with combined endpoint (new admission for heart failure, implantable cardioverter defibrillator implantation as a secondary prevention, and mortality) in patients with LVEF ≥ 30% (n = 46)

	Univariate		Multivariate	
	Odds ratios (95% CI}	p value	Odds ratios (95% CI)	p value
RVEF ≤ 45%	3.400 (0.195–59.374)	0.402		
LGE+	2.618 (0.655–10.453)	0.173		
FAC	0.890 (0.817–0.971)	0.008	0.890 (0.817–0.971)	0.008
LFT-RVFW	0.885 (0.797–0.984)	0.023	0.996 (0.841–1.179)	0.960

*RVEF* right ventricle ejection fraction, *LGE* late gadolinium enhancement, *FAC* fractional area change, *LFT-RVFW* longitudinal feature tracking-right ventricular free wall

## Discussion

The main findings of our study of CMR-FT in a population of patients with NICM were: a strong correlation between the calculation of RVEF by the classical method and LFT-RVFW/FAC derived from the FT technique; a higher probability of a combined event, as well as admissions for HF/mortality with lower values of LFT-RVFW/FAC in patients with LVEF ≥ 30% and preserved RVEF ≥ 45%.

Our group demonstrates with this study a strong correlation between the calculation of RVEF by the classical method of disc summation and the calculation of LFT-RVFW/CAF in patients with a diagnosis of NICM, which confirms the feasibility and robustness of the application of the FT method in this type of patient.

The predictive value of the RV function study is independent of left ventricular function in patients with HF and depressed LVEF [18, 19]. RV dysfunction is not only a marker of greater severity of left ventricular involvement and elevated pulmonary pressures, but also contributes to lower cardiac work efficiency, greater deterioration of functional capacity and development of HF [18, 19]. The prevalence of right ventricular dysfunction (RVEF < 45%) in NICM has been estimated in one third of patients, in agreement with our results (27.6%), and represents an independent marker of adverse events during evolution [19]. Thus, it has been shown that right ventricular function largely determines the prognosis of patients with NICM and HF. In this regard, in this population it has been estimated that patients with biventricular dysfunction, defined as LVEF < 35% and RVEF < 35%, present twice the risk of mortality than those with left dysfunction (LVEF < 35%), but with better right function (RVEF > 35%) [11].

In recent years, the assessment of RV myocardial deformation has gained importance as a marker of preclinical damage in various pathologies [20, 21]. The most common method of assessment is to determine RV myocardial deformation or strain by speckle tracking using TTE. However, since 2009 an analogous technique has emerged in CMR called feature tracking (FT), which allows the quantification of this parameter through sequences acquired in the usual routine such as cine b-SSFP sequences [21]. In the case of RV free wall longitudinal strain using speckle tracking by echocardiography, the cut-off points for prediction of RV dysfunction vary according to the authors. Focardi et al. [22] demonstrate that a cut-off point of < − 17% showed good agreement with an RVEF < 45%. At the same time, they find a good correlation of RV free wall longitudinal strain and RV FAC with CMR RVEF findings [22]. On the other hand, Lu et al. [23], establish a global RV longitudinal speckle tracking strain cut-off point of < − 20% to predict RVEF < 48%. Although RV CMR-FT assessment is a relatively recent and novel technique, normality values for the technique are available. Boyang et al. [24] studied a cohort of 100 healthy individuals; which included 10 men and 10 women from each 10-year age range between 20 and 70 years, finding LFT-RVFW values of − 23.9 ± 3.59% for men and − 24.6 ± 3.59% for women. On the other hand, Truong et al. [25], describe a LFT-RVFW normality value of − 22.11 ± 3.51% in a cohort of 50 healthy individuals. In relation to its ability to predict right ventricular dysfunction (RVEF < 45%), Tong et al. [26] establish as a cut-off point an LFT-RVFW of < − 24.4%. Comparing with the normal values and our findings for prediction of RVEF < 45% (LFT-RVFW < 18.5%, S = 0.86; E = 0.96), the very high value obtained by the latter group is striking, probably in relation to the established sensitivity of 100% and moderate specificity of 66.7%.

Regarding the development of HF, Carluccio et al. [27] compared global RV longitudinal strain with RV longitudinal free wall strain by echocardiography in a population with reduced LVEF, showing that only the latter was associated with the development of events during follow-up (12% mortality and 31% hospitalization due to HF decompensation) when corrected for left ventricular systolic function parameters. They concluded that, although both parameters have prognostic value, the consideration alone of RV longitudinal free wall deformation analysis (excluding the septum) represented a better predictor of events in patients with HF (cut-off point of < − 15.3%), mainly due to less influence of left ventricular longitudinal dysfunction. However, another group [28] found just the opposite, reporting that global RV longitudinal strain (cut-off point < − 17.3%) was a parameter that offered a better prediction of event development at follow-up compared to RV longitudinal free wall strain, but this was a population with functional tricuspid regurgitation and preserved LVEF. Given that our study population more closely resembles the first case, it was decided to select only LFT-RVFW, thus excluding septal values.

Consistent with the findings described, in our population we found that the group with the greatest benefit in the application of FT was the one with an LVEF greater than 30% and preserved RVEF (≥ 45%), in whom the disease was probably in its earlier stages. This, in our opinion, highlights the additional value of LFT-RVFW as an early marker of worse prognosis, allowing early intervention through more aggressive optimization of medical treatment. Thus, we consider that our results open a door for possible future multicenter studies with a larger number of patients that could corroborate our findings and consolidate the assessment of RV systolic function by CMR FT in patients diagnosed with NICM.

### Study strengths and limitations

Our study has several limitations. On the one hand, it is a retrospective study in which the identification of events was obtained by reviewing the electronic medical records, followed up by a telephone call, so that the loss of some of them during follow-up cannot be completely excluded. However, the incidence of lost cases was similar to that observed in other prospective studies [6]. Besides, limitations regarding the CMR-FT technique should also be mentioned. On the one hand, the temporal and spatial resolution is lower compared to echocardiography, although it has the advantage of not having acoustic window limitations. Likewise, the intra and interobserver variability must also be taken into account. It has been demonstrated that the intra and interobserver concordance of the CMR-FT technique is high, independently of whether it is evaluated in healthy individuals or in ones with cardiac pathology, with the FT-Longitudinal and circumferential being the most reproducible [21, 29].

## Conclusions

The evaluation of RV myocardial deformation by means of CMR FT in patients with NICM showed a strong correlation between LFT-RVFW and FAC values and RVEF measured by the classical method of disc summation, proving to be a feasible and robust technique. Moreover, in this population, LFT-RVFW and FAC values were associated with the incidence of the combined event (admission for HF, death, and ICD in secondary prevention) in patients without severe LV dysfunction and with preserved RVEF.

## Data Availability

The dataset for this present study is available from the corresponding author (javierurmeneta@hotmail.com) upon reasonable request.

## Abbreviations

BSA: Body surface area
CMR: Cardiac magnetic resonance
EDV: End diastolic volume
FAC: Fractional area change
FT: Feature tracking
HF: Heart failure
ICD: Implantable cardioverter defibrillator
LGE: Late gadolinium enhancement
LFT-RVFW: Longitudinal feature tracking-right ventricle free wall
LV: Left ventricle
LVEF: Left ventricular ejection fraction
NICM: Non-ischemic dilated cardiomyopathy
RV: Right ventricle
RVEF: Right ventricular ejection fraction
TTE: Transthoracic echocardiography
